# Learning based on patient case reviews: an interview study

**DOI:** 10.1186/1472-6920-8-43

**Published:** 2008-09-05

**Authors:** Rikke Sand Andersen, Rikke Pilegaard Hansen, Jens Søndergaard, Flemming Bro

**Affiliations:** 1 The Research Unit for General Practice, University of Aarhus, Århus, Denmark

## Abstract

**Background:**

Recent theories on adult learning recommend that learning is situated in real-life contexts. Learning is considered a continuous process in which every new experience builds on, and integrates with, previously accumulated experiences. Reviewing and reflecting on patient cases is in line with this learning approach. There has, however, been remarkably little research into how patient cases might be applied in professional education. The purpose of this article is to present family physicians' perceptions of the learning process initiated by reviewing patient cases.

**Methods:**

Thirteen family physicians, who had all participated in a large project on cancer diagnosis in family practice (the CAP-project), currently carried out at the Research Unit for General Practice, University of Aarhus were interviewed on their experiences of reviewing patient cases. In the CAP-project family physicians (n = 467, 81%) in the County of Aarhus (640 000 inhabitants) completed 2,212 (83%) detailed questionnaires on all newly diagnosed patients with cancer encountered in their practices during a one year period (2004–2005). In order to complete the questionnaire the family physicians were required to perform a systematic case review of each patient: they had to consult their records to provide dates of symptom-presentation, investigations and treatments initiated, and reflect on previous encounters with the patients to give detailed information on his/hers knowledge of the patients' care seeking behaviour, mental health and risk factors.

The purpose of this article is to present indebt interview-data on family physicians' perceptions of the learning process initiated by reviewing patient cases, and their evaluations of using patient case reviews as a learning method in family practice.

**Results:**

The process of reflection initiated by reviewing patient cases enabled family physicians to reconsider their clinical work procedures which potentially supported the transition from individual competence to personal capability. According to the physicians, they were not only able to identify needed changes, some reported that they were able to transform these ideas into action and do things more effectively. According to our data this transition takes place, because the learning processes initiated were based on real life experiences which equally initiated reflections on *what *to improve, as well as *how *to improve their work.

**Conclusion:**

Patient case reviews initiate reflective processes providing feedback about performance in real life situations. Family physicians are in favour of patient case reviews as a learning method, because it embraces the complexities they encounter in their daily practice and is based on personal experiences.

## Background

*Learning is the process by which experience is transformed into recognition *[1].

Family physicians are challenged to maintain their professional competences in an ever changing environment characterized by a continuing evidence expansion in their field [2,3]. This encourages educational providers to generate learning methods that equally develop the family physicians' general competences and their capabilities to adapt these to real life contexts [2,4].

Recent developments in adult learning theories, mainly inspired by complexity theory and experiential learning theories have taken up this challenge [1,2,5]. According to these theories focus on professional capability is best enhanced through process-oriented learning methods by which learning is driven by needs, is situated in real-life contexts, and allows time to reflect on own performances [2,4,6]. This means that learning is seen as a continuous process in which every new experience builds on, and integrates with, previously accumulated experiences. Reviewing and reflecting on patient cases is in line with this learning approach [4,7]. There has, however, been remarkably little research into how patient cases might be applied in professional education [4].

The study presented is part of a larger project on delay in cancer diagnosis in family practice (the CAP-project) currently carried out at the Research Unit for General Practice, University of Aarhus. 467 (81%) family physicians in the County of Aarhus (640 000 inhabitants) completed detailed questionnaires on 2,212 (83%) newly diagnosed patients with cancer encountered in their practices during a one year period (2004–2005) [8]. In order to complete the questionnaire the family physicians were required to perform a systematic case review of each patient: they had to consult their records to provide dates of symptom-presentation, investigations and treatments initiated, and reflect on previous encounters with the patients to give detailed information on his/hers knowledge of the patients' care seeking behaviour, mental health and risk factors.

Because the participating family physicians had systematically performed case reviews of the majority of all patients with cancer identified in their practices in 2004–2005, the CAP-project posed a unique opportunity to study the learning potentials of reviewing patient cases. We therefore decided to explore family physicians' experiences from the CAP-project through indebt interviews (Table 1). The purpose of this article is to present family physicians' perceptions of the learning process initiated by reviewing patient cases, and their evaluations of using patient case reviews as a learning method in family practice.

**Table 1:** Study design

**The CAP-study**
467 family physicians in the county of Aarhus completed 2,212 questionnaires in which they performed a systematic review of all newly diagnosed patients with cancer encountered in their practices during a one year period (2004–2005)

The main themes of the CAP questionnaire:
A description of the symptoms presented by the patients
Dates when patients first presented their symptoms to the family physician
Dates and descriptions of further investigations and treatments initiated
Responsibility for possible diagnostic delay in the health care system
A description of the family physicians' prior knowledge of the patient (care-seeking behaviour, mental health, risk factors)

**Study presented**
In order to study the learning potentials of reviewing patient cases thirteen family physicians from the CAP-study were interviewed on their perceptions of the learning processes initiated by reviewing patient cases, and their evaluations of using patient case reviews as a learning method in family practice

## Methods

### Setting

Denmark has a publicly funded health care system providing free access to family practice and hospital care. The family physicians function as gatekeepers to the rest of the health care system, carrying out initial investigations and referring patients to hospital or outpatient clinic treatment if necessary. Within an overall legal and logistic framework the individual family physicians' practices and work procedures are subject to internal variations.

### Participant selection

Our study is based on semi-structured, individual interviews. In order to obtain the broadest range of information and perspectives we selected family physicians according to gender, age and practice type. Initially we selected 18 family physicians from the CAP-database. Fourteen agreed to participate in the study; three participated in pilot interviews, one cancelled the interview due to illness, and ten were interviewed according to the final interview guide.

### Interviews

The first author (RSA), a trained anthropologist, conducted semi-structured interviews of approximately one hour duration with participating family practitioners. RSA was supervised by the co-authors, JS and FB, both family physicians and experienced in qualitative research methods. Before the interview each family physician received a written invitation to participate and subsequently gave verbal consent. To ensure reliability, the three first interviews were conducted during the development of the interview guide that was continually modified as new issues and themes emerged from the data. The interviews took place at the individual family physician's practice and were all tape-recorded. We conducted the interviews according to ethnographic principles for open-ended questions, meaning that the physicians were encouraged to speak freely and to raise issues of importance to them, also if the issues went beyond the topic-guide themes [9]. During the interviews, the family physicians were given paper copies of their CAP-questionnaires. Other studies have applied the same methods when exploring a similar setting [10].

### Data analysis

The interviews were transcribed verbatim by RSA, and all transcripts were read repeatedly to get an overall impression of the material before the initial coding. Initially, all meaningful text units in the transcripts were coded by theme [11]. Agreement was reached among the authors after thorough discussions of the initial coding. Subsequently, the themes were condensed and compared across groups, and main analytical categories were identified. During the last part of the analytic phase, theories of adult learning were used as an analytical basis for interpretation [1,2,5,6,12]. In order to clarify and link the different analytical categories with relevant theories, RSA wrote memos throughout the coding process [13]. We used the software package *NVivo, ed. 7 *to assist with coding, sorting and retrieval (Table 2).

**Table 2:** Analysis

We conducted a two-phased analysis:

**Phase one: Theme development.**
Themes developed:
1. Reactions to receiving questionnaires
2. Potential outcome
Diagnostic process
Evaluation of competences
Referral system
Awareness of delay
Organisational overview
3. Talk of change
4. Patient continuity
5. Family physician-patient relationship
6. Decision-making processes
7. Learning processes
Based on patient records
Based on daily experience
8. Learning by feedback
Experiences from other learning interventions
Positive and negative feedback
Learning from patient records
9. Clinical failures
All data were coded according to these themes in Nvivo ed. 7.

**Phase two: Development of analytical categories.**
The interviews as well as the code books of each identified theme were re-read.
The analytical categories were developed in a dialectic process between identified themes and the applied theoretical framework.
Analytical categories:
1. Learning processes described
Reflections on work procedures
Communication
Case manager function
2. Evaluation of patient case reviews
Patient-centeredness
Real life experiences

### Ethical approval

According to the Scientific Committee for the County of Aarhus, the Biomedical Research Ethics Committee System Act does not apply to this project. The study was approved by the Danish Data Protection Agency (J. nr. 2004-41-3705).

## Results

### Learning processes initiated by patient case reviews

The majority of the family physicians in our sample responded that reviewing patient cases gave rise to reflections on how their work was organized and carried out. They reported that the reflections resulted in more or less clear images of the structural and behavioural changes that were needed in their own practises, and it increased their capabilities to implement these changes, as it raised their awareness of how to navigate within the health care system. Two themes centred on the family physicians' case manager function and their communication strategies illustrate this.

#### Case manager

According to the family physicians, they are rarely able to predict their patients' clinical pathway when they are referred to hospital for further investigations, which impairs their case manager function.

"Looking back was good. We had lost contact with some of the patients. When we refer them [to the hospital] they often disappear from your system. It was good to see: well, what happened. It gives you something, it really does." (Informant 3, female, 25 years in family practice)

Reviewing patient cases gave them better insight into the time span and distribution of responsibility in relation to clinical pathways, thereby potentially improving their ability to perform this task. E.g., some family physicians stated that it had increased their attention on the delay patients experienced during hospital investigations. For some these insights had resulted in a change of strategy for dealing with delay and attempts to accelerate patients' pathway through the health care system:

"The questionnaires once again demonstrated that the time from referral to initial patient appointment and until the result of the examinations is known is long (...). You must try to keep this process as short as possible. Sometimes I ask the patients to help, to tell me if they wait too long. I think this [reviewing patient cases] has been a catalyst for me in making me do this even more." (Informant 5, male, 12 years in family practice)

To ask the patient to take on a more active role is just one in a series of strategies initiated to improve the family physician's case manager function. Many stated that they followed more pro-active referral procedures, such as phoning hospital departments to make appointments for their patients, or had become more selective in choosing which hospital departments or clinical investigations to refer to.

"I think we have to be very careful what we write in our referrals. If I suspect it is cancer, I have to write cancer. It has to b e very clear. Reviewing these patient cases has confirmed me in this." (Informant 9, male, 17 years in family practice)

#### Communication

The success of clinical pathways depends on the family physicians' communication skills and strategies at various levels: with patients, within the clinic and with hospital staff. According to the physicians reviewing patient cases may increase focus on all levels of communication.

"This [reviewing patient cases] makes you wiser professionally speaking, and it opens your eyes towards the weaknesses of our system, especially in relation to how we communicate with others. Sometimes they surprise you. 'Did this really take six months?"' (Informant 5, male, 12 years in family practice)

Individual family physicians described patient encounters where patients had failed to comply with their recommendations either by refusing further examinations or by failing to turn up at their appointments. In some of these cases lack of compliance was due to misunderstandings and to the fact that the family physician was not sure whether he or she had clearly indicated a suspicion of cancer.

"Well, she [the patient] didn't turn up for re-examinations as I had advised her to. And the fact that I had to go through her case again made me think if I had been too imprecise in my communications with her." (Informant 11, female, 23 years in family practice)

Reflections on these experiences had led to considerations about how to make their wording more precise, or how to ensure better continuity, e.g. by making sure patients keep future appointments and by explaining to the patient to contact the family physician again if symptoms become worse.

Reviewing patient cases also focused attention to communication within the clinic. In some partnership practices it resulted in spontaneous discussions about individual patients thus increasing the awareness of the beneficial use of group discussions:

"I believe that we consequently ... – well at least we do it more – discuss patients at our daily conference, if we think it went wrong, if things went too slow, or if we need to share experiences." (Informant 8, male, 18 years in family practice)

"Some of the questionnaires resulted in informal discussions during our breaks, and as such, they made us share experiences with each other in our practice." (Informant 1, female, 7 years in family practice)

For some, focus was turned towards the more specific procedures for handling external communication such as discharge letters, x-ray results and other test results. A family physician from a large practice told how incoming answers from hospitals were sometimes mislaid, and how they had previously considered how to improve their communication system to avoid this. Reviewing patient cases had convinced them that these changes were necessary and consequently the process had been accelerated.

**Figure 1:**
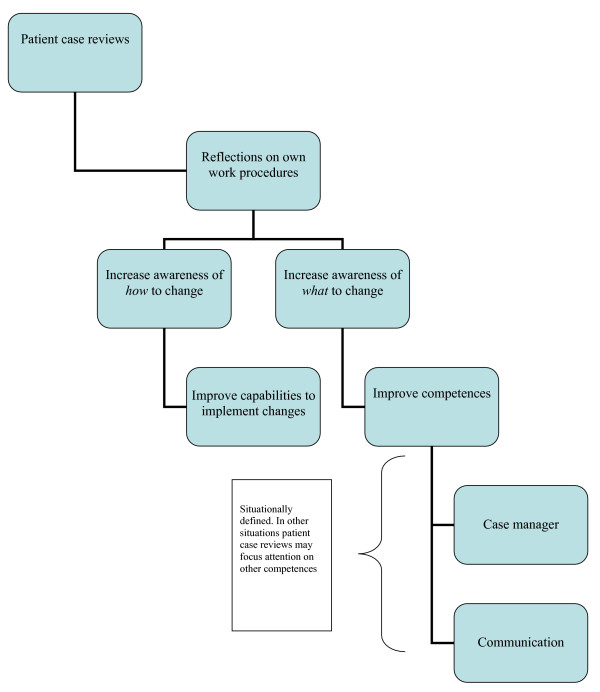
Learning process initiated by reviewing patient cases.

To summarize. The process of reflection initiated by reviewing patient cases enabled family physicians to reconsider their clinical work procedures which potentially supported the transition from individual competence to personal capability. According to the physicians, they were not only able to identify needed changes, some reported that they were able to transform these ideas into action and do things more effectively. According to our data this transition takes place, because the learning processes initiated were based on real life experiences which equally initiated reflections on *what *to improve, as well as *how *to improve their work (Figure 1).

### Family physicians' evaluations of patient case reviews as a learning method

Our data imply that the family physicians are in favour of using patient case reviews as a learning method. The fact that the patient cases are patient-centred and based on the family physicians' real-life experiences highly increase the level of motivation to take up learning.

#### Patient-centred

When asked about their perceptions of using patient case reviews as a learning method, the family physicians made a variety of emotional responses such as "it feels very intrusive" and "it touches me as a human being, which makes it really efficient." Our data suggest that this is due to the fact that family physicians perceive of their clinical work as embedded in social and moral obligations. They do not have a conclusive view on their patients as 'just patients', and their clinical work does not take place in a vacuum, but is part of a wider social and professional framework, according to which they evaluate their work. According to the family physicians the fact that they were reviewing their own patients potentially improved the impact of learning.

"The examples always stand stronger when they are 'in flesh and blood', so to speak. I always listen to the patient stories of my colleagues, but of course, when I am faced with the stories of the patients I know personally it has a stronger effect on me. This man for example, I knew his family. That means something." (Informant 1, female, 7 years in family practice)

"It is more relevant, simple because it is my own patients. I like that." (Informant 2, female, 20 years in family practice)

Some of the family physicians' responses suggest that the fact that the patient cases reviewed were cancer-related, and thus often long-term and severe, increased the learning impact of the patient case reviews.

"Well, these patients they have really made an impression on you, because you come to know them and their families. And then when they die....Of course that concerns me and my colleagues. But that was the powerful element in going through these cases again." (Informant 7, female, 9 years in family practice)

#### Real life experiences

The family physicians were also in favour of using patient case reviews as a learning method because they embrace the complexity they experience in their daily practice. Various factors such as age, medical history, physician-patient relation etc. influence their work, and no patients are alike.

"When we see a classic case, one that fits the descriptions in the medical textbooks, we tell each other because it is so rare." (Informant 5, male, 12 years in family practice)

"The good thing about focusing on your own patients is that focus is on *how do you make things happen *in real life, more than just information on how to do this and this. When I look back at my own work and my own experiences it becomes a kind of inspiration to me: what can I do in order to improve my work." (Informant 12, male, 7 years in family practice)

Another aspect of this was that family physicians expressed that the process of 'looking back' and reviewing individual patient cases was well-known to many of them. When talking in abstract terms about their work, and when referring to their clinical experiences such as establishing a cancer diagnosis, many family physicians said that the management of patients is often based on clinical experiences of similar cases. However, they welcomed a means of systematising this part of their job:

"I often look back. What did I do? When did I do what? But this [reviewing patient cases] was good, more systematic. And when you act on your own initiative, perhaps you only see what you want to see. This made you go through the whole process." (Informant 1, female, 7 years in family practice)

## Discussion

Our study illustrates that reviewing patient cases has a learning potential because it initiates a reflective process that equally provide feedback about performance in real life situations and potentially enhance family physicians' capabilities implement changes. It also demonstrates that family physicians are in favour of this method, as it embraces the complexities they encounter in their daily practice (Table 3).

**Table 3:** What this study adds

**What is already known:**
Learning is best enhanced through process-oriented learning methods where learning is driven by needs, is situated in real-life contexts, and allows time to reflect on own performances.

**What this study adds:**
Reviewing patient cases allowed family physicians to reconsider their clinical work procedures, which enabled them to identify needed changes, as well as to transform these into action and do things more effectively.

**Suggestions for future research:**
Learning based on patient cases represents real life dilemmas and it is inevitably based on the subjective experiences of the participating physicians. Therefore, the future challenge is to develop methods that ensure reliable learning from the experiences of daily practice.

Because of its qualitative perspective the study provides insights into the family physicians' subjective opinions, expectations and motivations and give in depth descriptions of potential learning processes initiated by patient case reviews. However, our results are based on family physicians' self-reported perceptions which call for further empirical studies, as our data do not allow us to evaluate whether changes were actually implemented. Finally our results may be biased toward a positive view on reviewing patient cases, because the participating physicians were selected among participants from the CAP-project, who had indirectly taken a positive interest in reviewing patient cases.

A growing list of publications confirm that it is advantageous to apply learning methods that are structured around individual experiences because it increases the learners' potentials for acquiring new competences and improve their ability to apply these in daily practice [2,4,12,14-18]. The development of methods complying with this learning approach has been defined as a future challenge to continuing medical education [4]. Our study shows that reviewing patient cases initiated a learning process that may meet the required demands, as they made family physicians reflect on work procedures, identify needed changes, and raised their awareness of how to implement changes.

Another theme addressed in the paper was the family physicians' perceptions of using patient case reviews as a learning method. Our paper demonstrates that family physicians are in favour of applying patient case reviews as a learning method, because they embrace the complexities they encounter in their daily practice. Common claims against traditional learning methods have been that they are 'check-list driven' and fail to recognise the complex context into which new knowledge is integrated [19,20]. Our study indicate that reviewing patient cases evoke the complexity of the clinical situation and potentially assist in paying attention to how 'new knowledge' integrates with complex clinical reasoning [21].

However, learning based on patient cases represents real life dilemmas and it is inevitably based on the subjective experiences and decisions of the participating physicians. Therefore, the future challenge to educators and physicians is to develop methods that ensure reliable learning from the experiences of daily practice. Conducting patient case reviews in groups with a facilitator or in an audit-group may improve the likelihood of a fruitful outcome, and hence improve the level of quality assurance.

## Conclusion

Our results illustrate that patient case reviews initiate reflective processes providing feedback about performance in real life situations. It also demonstrates that family physicians are in favour of patient case reviews as a learning method, because it embraces the complexities they encounter in their daily practice and is based on personal experiences.

## Competing interests

The authors declare that they have no competing interests.

## Authors' contributions

RSA has made contributions to the study design, has been in charge of the acquisition of data, contributed to data-analysis and written the final manuscript.  JS has contributed with analysis and interpretation of data, critically revised the article for important intellectual content, and read and approved the final version of the manuscript.  RPH has contributed with analysis and interpretation of data, critically revised the article, and read and approved the final version of the manuscript.  FB Has made substantial contributions to the design of the study, interpretation of data, critically revised the article for important intellectual content and read and approved of the final version of the manuscript.

## Pre-publication history

The pre-publication history for this paper can be accessed here:


